# Diffusion Basis Restricted Fraction as a Putative Magnetic Resonance Imaging Marker of Neuroinflammation: Histological Evidence, Diagnostic Accuracy, and Translational Potential

**DOI:** 10.3390/life15101599

**Published:** 2025-10-14

**Authors:** Szabolcs Kéri

**Affiliations:** 1 Sztárai Institute, University of Tokaj, 3944 Sárospatak, Hungary; keri.szabolcs@unithe.hu; 2 Department of Physiology, Albert Szent-Györgyi Medical School, University of Szeged, 6722 Szeged, Hungary

**Keywords:** magnetic resonance imaging, diffusion-based imaging, neuroinflammation, multiple sclerosis, neurodegeneration, mood disorders

## Abstract

Diffusion basis spectrum imaging–derived restricted fraction (DBSI-RF) isolates the low apparent diffusion coefficient water signal attributed to cellular crowding. It is therefore proposed as a putative magnetic resonance imaging (MRI) marker of neuroinflammation. The purpose of this narrative review is to evaluate animal and human studies that compared DBSI-RF with histopathological benchmarks and clinical parameters. Across inflammatory demyelination, viral encephalitis, traumatic brain injury, and neurodegenerative disorders, DBSI-RF correlated moderately to strongly with immune cell density and distinguished inflammation from demyelinating or axonal pathology. In acute multiple sclerosis, combined isotropic fractions predicted lesion evolution, clinical subtypes, and deep-learning models that included DBSI-RF classified lesion subtypes with high accuracy. DBSI-RF might also be used to track putative neuroinflammation associated with psychosocial stress, mood disorders, and anxiety disorders. The strengths of the method include sensitivity to subclinical changes and the concurrent mapping of coexisting edema, demyelination, and axon loss. Limitations include non-specific etiology features, a demanding acquisition protocol, and limited large-scale human validation. Overall, DBSI-RF may demonstrate a promising diagnostic and prognostic accuracy, warranting standardized, multicenter, prospective trials and external validation.

## 1. Introduction: Diffusion Imaging and Neuroinflammation

Neuroinflammation, the activation of immune cells and associated molecular cascades in the central nervous system, is increasingly recognized as a key factor in several neurological and psychiatric disorders. Classic neuroinflammatory diseases like multiple sclerosis (MS) and traumatic brain injury (TBI), and neurodegenerative disorders such as Alzheimer’s disease (AD), as well as metabolic and psychiatric conditions (e.g., obesity, mood disorders, and psychotic disorders), all involve neuroinflammatory processes [1,2,3,4,5,6].

Accurate and noninvasive brain biomarkers of neuroinflammation could enhance diagnosis, disease monitoring, and evaluation of therapeutic responses. Conventional magnetic resonance imaging (MRI) and even standard diffusion tensor imaging (DTI) struggle to specifically identify neuroinflammation, since imaging abnormalities (e.g., T_2_ lesions or reduced fractional anisotropy) can result from various co-occurring pathologies (demyelination, axonal injury, edema, or inflammatory cell infiltration) [7,8]. Positron emission tomography (PET) with 18 kDa translocator protein radioligands can directly visualize activated microglia. Still, PET is costly, involves radiation, and has yielded mixed results in detecting inflammation [9].

**Figure 1:**
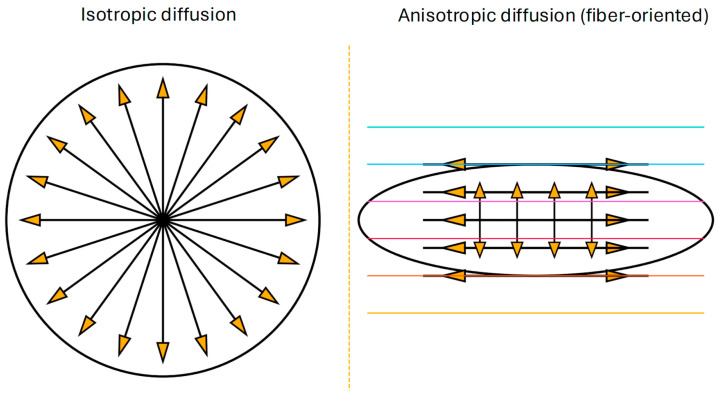
Illustration of isotropic and anisotropic diffusion. Isotropic diffusion: A central point emits equal-length arrows in all directions inside a circular boundary, which represents direction-independent displacement of water diffusing freely in a homogeneous medium. In tensor terms, this corresponds to a spherical diffusion tensor with equal eigenvalues (λ_1_ ≈ λ_2_ ≈ λ_3_). There is no preferred direction, and fractional anisotropy (FA) = 0. Anisotropic diffusion: An elongated ellipse represents a displacement distribution with a significantly greater spread along one axis than perpendicular to it. Parallel lines illustrate a fiber bundle. Long, bidirectional arrows run along the fiber axis (high axial diffusivity, λ_1_), while short arrows perpendicular to the fiber indicate low radial diffusivity (λ_2_, λ_3_). Here, the diffusion tensor is ellipsoidal with a clear principal eigenvector aligned to the fiber, and FA > 0.

Diffusion basis spectrum imaging (DBSI) is an advanced diffusion MRI technique that decomposes the water diffusion-weighted signal into multiple components, distinguishing anisotropic fiber diffusion (axons) from various isotropic diffusion components (Figure 1) [10,11].

**Figure 2:**
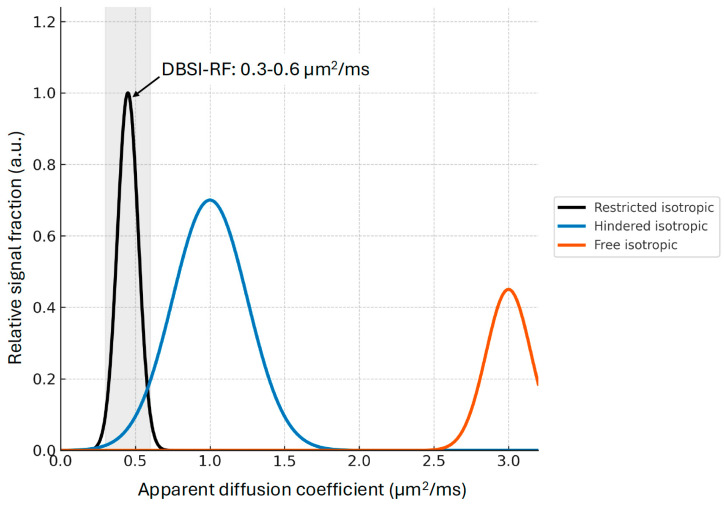
Restricted isotropic diffusion fraction (DBSI-RF). DBSI-RF is typically defined within an apparent diffusion coefficient band of 0.3–0.6 μm^2^/ms and is interpreted as the volume fraction of water trapped by cells (e.g., inflammatory cells), thereby distinguishing it from hindered and free water fractions.

In DBSI, water diffusion in cellular structures is modeled as a restricted isotropic diffusion fraction (DBSI-RF), typically defined by very low apparent diffusion coefficients (0.3–0.6 μm^2^/ms) (Figure 2). This restricted fraction (RF) is interpreted as the volume fraction of water trapped by cells (e.g., inflammatory cells).

**Figure 3:**
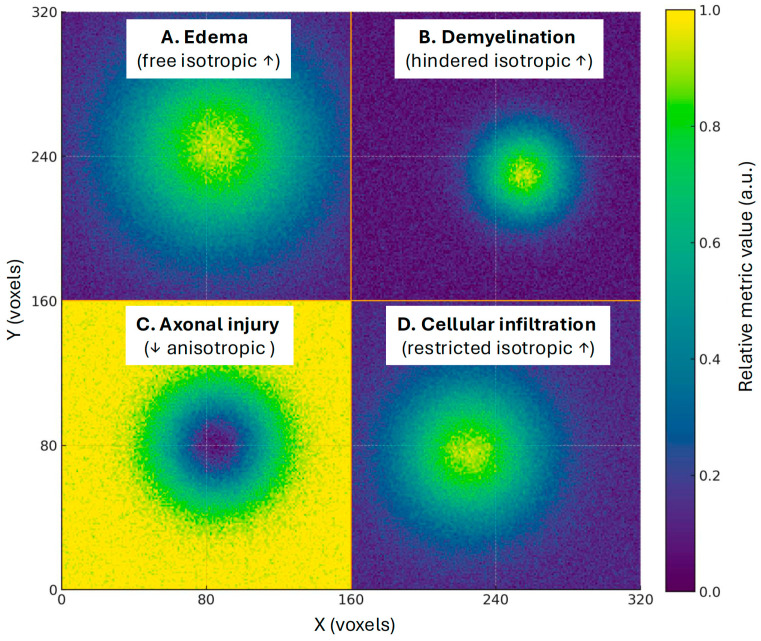
Schematic illustration of potentially coexisting tissue processes in distinct parametric maps. Each panel represents a different DBSI-derived metric rendered as a heat map. Brighter values indicate a higher relative metric value, while darker values indicate a lower relative metric value. (**A**). Edema (free isotropic ↑): broad, bright area reflecting elevated free-water content. Extracellular fluid accumulation (vasogenic edema) raises the fast, high apparent diffusion coefficient component. DBSI quantifies this as increased free isotropic diffusion. (**B**). Demyelination (hindered isotropic ↑): a moderately brighter region signifies an increase in hindered isotropic diffusion. Loss of myelin sheaths and microstructural disorganization cause water to shift into less-restricted, tortuous spaces. DBSI captures this as a rise in the hindered isotropic compartment. (**C**). Axonal injury (↓ anisotropic fiber fraction): a darker focal region in the center-right of this panel indicates a local reduction in the anisotropic (fiber-directed) component, consistent with axonal loss or injury. DBSI models orient axonal diffusion separately from isotropic water. A drop in the anisotropic fraction suggests fewer intact, coherently oriented axons. (**D**). Cellular infiltration (restricted isotropic ↑): a more focal bright spot reflecting an increased restricted fraction. Infiltrating inflammatory cells and activated glia increase cell density, trapping water and boosting the restricted isotropic compartment used as a putative MRI marker of neuroinflammation.

By separating RF from hindered diffusion (characterized by a moderate apparent diffusion coefficient, indicating less densely packed environments) and free diffusion (characterized by a high apparent diffusion coefficient, e.g., edema and extracellular water), DBSI can quantify coexisting pathologies such as axonal injury, demyelination, edema, and cellular infiltration (Figure 3).

This technique aims to overcome the limitations of conventional DTI, which cannot distinguish between reduced diffusion due to cell influx and other factors, such as myelin loss or edema [12,13,14]. Nevertheless, DTI is widely accepted in medical research due to its fast acquisition protocol, which is particularly practical for studying neurodegenerative diseases in patients with associated movement disorders, where motion artifacts are common [15]. DBSI calculations in most studies are based on multi-shell acquisition methods, which often require multiple b-values and many diffusion directions, i.e., acquisitions that are more demanding than standard mono-shell DTI. Therefore, the direct comparison of DTI and DBSI is problematic. Overall, DBSI-RF is hypothesized to serve as an MRI marker of neuroinflammation, since cellular infiltration and glial activation in neural tissue increase the density of cells. Thus, the fraction of the restricted diffusion compartment is also elevated [16,17,18,19,20,21].

It is worth noting that various other diffusion MRI techniques can supplement DSBI-RF. For example, free-water imaging (FWI) indexes extracellular water and has been tied to inflammation and degeneration in AD and other disorders. It is often increased where microglial activation is present [22]. Second, neurite orientation dispersion and density imaging (NODDI) estimates neurite density and orientation dispersion, a measure of axonal and dendritic integrity. It has been shown to relate to amyloid-beta and tau pathology prior to cortical thinning in AD [23]. Third, the soma and neurite density image (SANDI) explicitly models soma and neurites and is feasible for cortex and high-gradient systems [24]. It could complement DSBI-RF for distinguishing between “cellularity” and “neurite loss”. Indeed, recent studies argue that multi-model imaging syntheses can better map neuroinflammatory processes than any single model [25].

When considering the usefulness of DBSI-RF, we should first evaluate the relationship between the DBSI-RF signal and the histological features of neuroinflammation. Second, it is essential to investigate its diagnostic performance and predictive value in clinical studies. The purpose of this narrative review is to delineate these questions, illustrating the relevance of new diffusion MRI techniques in transdiagnostic clinical assessment.

## 2. Methods

The principles of Scale for the Assessment of Narrative Review Articles (SANRA) was used for the standards of the present narrative review: “(1) justification of the article’s importance for the readership, (2) statement of concrete aims and formulations of questions, (3) description of the literature search, (4) referencing, (5) scientific reasoning, and (6) appropriate presentation of data” [26]. We had three Scope and Key Questions (KQs): KQ1 (criterion validity): How does DBSI-RF relate to histological markers of cellularity in animals and post-mortem human samples? KQ2 (diagnostic/prognostic performance in humans): What is the incremental value of DBSI-RF versus conventional MRI/DTI for lesion characterization or clinical outcomes? KQ3 (translational/psychiatric and metabolic contexts): What exploratory associations link DBSI-RF with symptoms or treatment response?

We searched the following databases up to August 2025: Scopus, Web of Science, PubMed, and Semantic Scholar. The search keys included “diffusion basis spectrum imaging”, “DBSI”, “restricted fraction”, and “neuroinflammation”. The reference lists of the obtained papers were manually screened to find relevant articles. We used a dual independent screening and extraction process, with two reviewers. Discrepancies were resolved through consensus. In addition to the author, an independent reviewer naïve to the aim of the study assessed the search process and the suitability of papers according to the KQs. The independent reviewer also screened the paper using the SANRA criteria.

The findings were synthesized, organized and presented according to the following narrative scheme: (1) the correlation between DBSI-RF and histopathological markers of neuroinflammation: animal models of inflammatory myelin pathology, traumatic brain injury models, autopsy from human spinal cord, and neurodegeneration; (2) diagnostic performance of DBSI-RF in clinical samples: lesion characterization in MS, obesity and metabolic syndrome, and neuropsychiatric disorders; (3) technological strengths and limitations of DBSI-RF.

## 3. Correlation of DBSI-RF with Histopathological Markers

### 3.1. Animal Models of Inflammation and Demyelination

In a mouse study of inflammatory demyelination (the cuprizone model of MS), DBSI-RF closely followed cellular infiltration. DBSI-derived “cellular” fraction strongly correlated with histological cell counts (4′,6-diamidino-2-phenylindole [DAPI]-positive nuclei) in white matter [10]. This high correlation provided early evidence that the restricted isotropic component quantitatively reflects increased cellularity in tissue (Table 1).

Multiple mouse studies employing experimental autoimmune encephalomyelitis (EAE) and optic neuritis have demonstrated robust correlations between DBSI metrics and immunohistochemistry [17,27]. In optic neuritis, the DBSI restricted fraction increases with the number of DAPI-counted nuclei and decreases with anti-inflammatory treatment. In contrast, DBSI fiber fraction declines in proportion to axonal loss measured by SMI-312 or neurofilament stains [28]. A longitudinal EAE optic neuritis study using dexamethasone showed expected short-term reductions in inflammation but no durable protection from axonal loss, and voxel-wise scatter plots confirmed direct correlations between DAPI counts and DBSI-RF and inverse relations between DBSI-RF and axonal markers [29] (Table 1).

Finally, Zhan et al. (2018) assessed hippocampal CA1 lesions, including neuronal dendritic injury and concomitant inflammation, in Theiler’s murine encephalomyelitis virus-induced seizure mice [30]. DBSI-RF correlated with DAPI-positive nucleus counts, and DBSI-derived fiber fraction correlated with dendrite density. The results also revealed that DTI-FA was less specific than DBSI-derived pathological metrics for hippocampal CA1 dendritic injury and inflammation [30].

**Table 1:** Studies investigating the histological correlates of tissue diffusion properties.

Study	Target	Sample	Condition/MRI	Validation	Correlation with Histology
Wang et al., 2015 [11]	Axonal loss, myelin loss, inflammation (restricted fraction, fiber fraction, radial diffusivity)	3 autopsy, 5 controls, 6 multiple sclerosis patients	Multiple sclerosis 3T TIM Trio (Siemens, Munich, Germany ) voxel: 2 × 2 × 2 mm^3^	Bielschowsk silver, H&E, Luxol fast blue-PAS; cervical spinal cord	Fiber fraction vs. silver: r = 0.70–0.83; radial diffusivity vs. Luxol fast blue: r = −0.84 to −0.42; restricted fraction vs. H&E: r = 0.84–0.39
Wang et al., 2011 [10]	Cellularity (restricted fraction), axonal/ myelin injury	5 mice/group	Cuprizone-induced demyelination 4.7T Varian DirectDrive (Palo Alto, California) 0.75 mm slice thickness, 128 × 128 data matrix (70 × 70 µm voxel dimension)	SMI-31, MBP, DAPI; corpus callosum	Cell ratio vs. DAPI: r = 0.76; axial diffusivity vs. SMI-31: r = 0.76; radial diffusivity vs. myelinated axon: r = −0.76
Wang et al., 2014 [27]	Axonal injury, myelin integrity, inflammation (restricted fraction)	5 mice/group	Experimental autoimmune encephalomyelitis 4.7T Agilent DirectDrive (Santa Clara, California) 1 mm slice thickness, 128 × 128 data matrix (70 × 70 µm voxel dimension)	DAPI, SMI31, myelin basic protein; spinal cord ventrolateral white matter	Restricted fraction vs. DAPI: r = 0.90; radial diffusivity vs. MBP: r = −0.78; parallel diffusivity vs. SMI31: r = 0.74
Zhan et al., 2018 [30]	Dendritic injury, inflammation (restricted fraction, fiber fraction)	5 mice/group	Theiler’s murine encephalomyelitis virus-induced hippocampal inflammation 11.74T Agilent DirectDrive (Santa Clara, California) 0.5 mm slice thickness, 192 × 192 data matrix	NeuN, MAP2, IBA1, DAPI; hippocampus	Restricted fraction vs. DAPI: r = 0.81; fiber fraction vs. MAP2: r = 0.79; FA vs. MAP2: r = 0.78; FA vs. DAPI: r = −0.82
Lin et al., 2017 [17]	Axonal loss, demyelination, inflammation	8 mice	Experimental autoimmune encephalomyelitis optic neuritis 4.7 T Agilent DirectDrive (Santa Clara, California) 1 mm slice thickness, in-plane resolution: 117 μm^2^	SMI312, SMI31, MBP; optic nerve	Restricted fraction vs. DAPI: r = 0.99, fiber fraction vs. SMI312: r = 0.85

FA—fractional anisotropy; H&E—hematoxylin-eosin; Iba1—ionized calcium-binding adapter molecule 1; MAP2—microtubule-associated protein 2; MBP—myelin basic protein; NeuN—neuron-specific nuclear protein; PAS—periodic acid-Schiff; SMI31—Sternberger Monoclonals, Inc., monoclonal antibody that detects phosphorylated neurofilament H; SMI312—monoclonal antibody, pan-axonal marker for neurofilaments; DAPI—4′,6-diamidino-2-phenylindole.

### 3.2. Animal Models of TBI

Neuroinflammation is also a key feature of TBI. In a mouse closed-head TBI model focusing on traumatic optic neuropathy, DBSI was used to detect subclinical optic nerve pathology. Imaging-histology correlations showed that elevated DBSI-RF corresponded to increased cellularity in injured optic nerves. This suggests a substantial association between the MRI metric and histological inflammatory cell presence [20], which is consistent with data from human patients [31].

Likewise, in a rat spinal cord injury model (rhizotomy leading to Wallerian degeneration), the fraction of extra-axonal restricted diffusion (conceptually analogous to DBSI-RF) showed high correlations with activated microglia density (Iba-1 staining) in the dorsal columns [32]. This provided the first histological evidence that increased restricted diffusion arises from microglial and macrophage invasion in injured white matter.

### 3.3. Autopsy from Human Spinal Cord in Inflammatory Demyelination

A study co-registering post-mortem MS spinal cord lesions with MRI demonstrated that DBSI-RF maps histological inflammation [11] (Table 1). In three autopsy specimens, DBSI-RF correlated with the area of immune-cell nuclei in lesion voxels for those cases with substantial inflammation. In one specimen with heavy inflammatory infiltrates, the correlation was very high (r = 0.84, *p* < 0.0001). A second specimen with scant inflammatory cells showed no significant correlation (r = 0.25, *p* = 0.23), and a third with modest inflammation had a moderate correlation (r = 0.39, *p* = 0.033) [11]. The authors attributed the absent or weak correlations in the latter cases to the low cellular infiltration, noting that where inflammation was minimal, the DBSI-RF showed less correspondence. Importantly, other DBSI metrics validated specificity for different pathologies (e.g., fiber fraction vs. axonal content, radial diffusivity vs. myelin loss), reinforcing that DBSI-RF specifically tracked the cellular component [11].

A separate brief report later compared in vivo DBSI maps with biopsy results from an inflammatory demyelinating brain lesion, showing a pattern of increased DBSI radial diffusivity, an elevated non-restricted fraction, a reduced fiber fraction, and preserved axial diffusivity, mirroring demyelination with relative axonal sparing on histology [19]. Although single-case and descriptive, this study provided a rare direct alignment between imaging and histology in a living patient.

### 3.4. Neurodegenerative Diseases

Preliminary studies suggest that DBSI-derived metrics can putatively capture neuroinflammation in diseases like AD [33]. Ex vivo DBSI on human AD brain tissue, combined with immunohistochemical staining of microglia (Iba-1) and computational modeling, has shown increased RF in white matter compared to controls, aligning with microglial activation and cellular debris in AD [34]. These early findings, along with rodent models of AD [35], indicate a potential for DBSI-RF to quantify neuroinflammatory components in neurodegenerative disorders. However, further research and direct histopathological validation are needed. For example, chronic manganese (Mn) neurotoxicity in welders with Mn exposure was assessed using DBSI: Mn-exposed individuals showed greater DBSI-RF in the white matter, consistent with neuroinflammation and Mn-related microstructural changes, which may be implicated in various neurodegenerative diseases [35,36,37].

### 3.5. Summary of Histopathological Evidence

Across diverse models, from autoimmune demyelination to TBI, DBSI-RF consistently mirrors the magnitude of cellular infiltration measured in tissue (Table 1). Correlation coefficients between the DBSI-RF and quantitative histology range from 0.7 to 0.9 in high-inflammation settings, underscoring a strong concordance. In cases of milder inflammation, DBSI-RF shows only mild or no elevation, corresponding to weaker correlations with histology. This body of evidence supports the validity of the DBSI-RF as a potential biomarker of neuroinflammation, with histopathology serving as the reference standard. However, further studies are needed to reveal specificity and sensitivity. Additionally, the discrepancy between MRI voxel size and the optical resolution of histopathological imaging may be a limiting factor in making direct and accurate correlations.

## 4. Diagnostic Performance and Predictive Value

### 4.1. Lesion Identification, Classification, and Outcome in MS

An essential aspect of evaluating DBSI-RF is its diagnostic accuracy, which refers to how sensitively and specifically it can detect or predict neuroinflammatory pathology, as well as its utility in clinical and research settings. Several studies have examined the performance of DBSI-derived metrics (including RF) in distinguishing pathological states or predicting outcomes, often in comparison to conventional MRI or DTI metrics, yet the results are still preliminary.

DBSI has demonstrated an improved ability to characterize MS lesions [16,38]. Using multiple diffusion metrics (including RF) as inputs, deep learning classifiers achieved high accuracy in identifying lesion pathology subtypes. In one study, a neural network combining DBSI metrics correctly classified MS lesion types with an overall concordance of 93.4%. These models outperformed those using only DTI or conventional MRI features, which achieved 74–80% accuracy [39]. Critically, the DBSI-based model yielded the highest sensitivity and specificity in differentiating lesions with high inflammation vs. those dominated by demyelination or axonal loss [39].

Initial evidence suggests that DBSI-RF may have prognostic value in MS. A recent study evaluated acute MS lesions and their evolution into chronic “black holes” (persistent T1-hypointense lesions indicating severe tissue damage) [12]. DBSI isotropic fractions, both restricted (cellular) and non-restricted (edema), were significant predictors of which acute lesions became persistent “black holes” at 12 months. This implies that DBSI metrics (especially when capturing higher cellular inflammation) might serve as early indicators of aggressive lesion pathology. Traditional DTI metrics alone did not predict outcome as well, highlighting that DBSI-RF added predictive value by gauging the inflammatory aspect of lesions that contribute to subsequent neurodegeneration [12].

In a cross-sectional study of 103 patients with clinically isolated syndrome, relapsing–remitting MS, secondary progressive MS, and primary progressive MS, T1/T2 relaxation and DBSI metrics were assessed in whole brain, normal-appearing white matter, lesions, and corpus callosum to index neuroinflammation, demyelination, and axonal injury [40]. MRI metrics showed a stepwise worsening from clinically isolated syndrome to relapsing-remitting MS to progressive MS. Relapsing-remitting MS was characterized by prominent edema-related abnormalities. In contrast, progressive MS exhibited the most extensive myelin and axonal damage [40]. Overall, relaxation and DBSI-derived measures differentiated MS subtypes by both severity and composition of tissue injury. The severity can also be characterized in terms of non-motor symptoms in MS. DBSI applied to the normal-appearing corpus callosum linked reduced fiber fraction and increased non-restricted fraction to worse cognitive performance, supporting its sensitivity to clinically relevant axonal loss and edema [38].

### 4.2. Obesity and Neuroinflammation

In conditions like obesity, where inflammation is relatively subtle and diffuse, DBSI-RF has demonstrated sensitivity to group differences. In two cohorts of obese vs. lean adults, the obese groups had significantly higher DBSI-RF values in multiple white matter tracts [18,41]. The DBSI-RF increases were interpreted as evidence of obesity-linked putative neuroinflammation. These alterations in the hippocampus and amygdala were linked to cognitive performance [18], which is consistent with animal models and molecular evidence [42,43]. DBSI-RF also provides information about adipose tissue distribution. Higher DSBI-RF and lower axial diffusivity were observed in widespread white matter in adults with obesity with higher subcutaneous and visceral abdominal fat, especially in females [44].

In pediatric obesity, DBSI findings corroborated these results. Children with weight problems exhibited higher putative MRI neuroinflammatory markers in the white matter, and diffusion metrics, such as DBSI-RF, could distinguish obese from normal-weight children with good sensitivity and specificity [41]. Specifically, in 601 children, overweight or obesity was associated with widespread white matter DBSI-RF increases and higher RF in the hypothalamus and striatum, which correlated with body mass index and waist circumference. DBSI-RF also paralleled restriction spectrum imaging findings and classified obesity status similarly, whereas baseline hypothalamic DBSI-RF only nominally predicted a 2-year increase in waist circumference. Interestingly, these data provided information about the neuroanatomical correlates of obesity, including satiety regulation in the hypothalamus, and reward associated with food-related cues in the striatum and nucleus accumbens [41,45].

Taken together, current evidence suggests that DBSI-RF is a potentially sensitive and indirect marker of obesity-associated neuroinflammation. The metric aligns with restriction spectrum imaging findings, but it remains an imaging surrogate rather than a direct histological readout (albeit supported by validation of DBSI metrics in other contexts). The longitudinal predictive effects of DBSI-RF on future changes in adiposity are small, underscoring the need for interventional and mechanistic studies.

### 4.3. Neuropsychiatric Disorders

Inflammation mechanistically links obesity and metabolic syndrome with AD, depression, and other mental disorders [46], which supports the potential transdiagnostic relevance of DBSI-RF. Regarding the cellular and molecular bases, adipocyte hypertrophy promotes macrophage infiltration and the secretion of proinflammatory cytokines (e.g., interleukin-1β, interleukin-6, and Tumor Necrosis Factor-α). At the same time, saturated fatty acids activate Toll-like receptor-mediated signaling, a key factor in regulating immune cells. Meanwhile, stress-related hyperactivation of the hippocampal-pituitary-adrenal axis drives glucocorticoid-related neuronal injury. Ultimately, obesity and neuropsychiatric disorders are associated with increased oxidative stress, impaired neurogenesis and synaptic plasticity, loosened blood–brain barrier tight junctions, and proinflammatory microglial phenotypes, characterized by reduced amyloid-beta (Aβ) clearance. Insulin resistance feeds back via insulin-degrading enzyme-dependent Aβ handling and tau phosphorylation, increasing the risk of neurodegeneration and AD. Finally, gut dysbiosis may further amplify these cascades [46,47,48,49].

In a preliminary longitudinal study, Wang et al. (2016) [50] found a strong association between PK11195 PET images (a measure of microglia activation) and DBSI-RF in individuals who developed mild cognitive impairment, a high-risk condition for AD. Moreover, DBSI-RF increased over the course of the follow-up period in Aβ-positive individuals who developed AD. More recently, Sathe et al. (2024) reported that hippocampal free-water explained 8.13% of the variance in memory decline over a period of up to 5 years in a large population of elderly individuals with and without mild cognitive impairment [51]. Advanced models using deep learning algorithms should consider DBSI-RF in the prediction of cognitive decline in AD as part of a multimodal assessment, including neuropsychological tests, imaging data, and peripheral plasma biomarkers (e.g., Aβ42/β40, phosphorylated tau, and neurofilament light) [44].

A new application area is Parkinson’s disease, where DBSI-RF detects microstructural changes in both the substantia nigra and white matter tracts. Early-stage Parkinson’s patients show significantly higher DBSI-RF in the substantia nigra compared to healthy controls, indicating inflammatory cell infiltration [37]. This is accompanied by reduced fiber fraction (loss of axonal/dendritic density) and increased non-restricted fraction (edema or extracellular space expansion). Importantly, DBSI measures in the substantia nigra and other brain regions correlate with neuropsychiatric symptoms, such as anxiety and cognitive performance [37]. Critically, no such associations are found when conventional DTI fractional anisotropy is analyzed. This suggests that DBSI can differentiate inflammatory from degenerative components in Parkinson’s, offering a potential tool for tracking disease progression before the overt decline of motor symptoms.

Large-scale studies have started to link putative neuroinflammation markers to clinical outcomes in mental disorders. In one analysis of over 11,000 individuals from the UK Biobank, higher DBSI-RF in the amygdala was associated with recent depression, mediated by low-grade peripheral inflammation (C-reactive protein) [52]. A predictive model using amygdala DBSI-RF for depression achieved modest performance (specificity: 0.62, sensitivity: 0.56), suggesting some predictive value, though not strong enough alone for clinical diagnosis in the general population [52]. Supporting its transdiagnostic nature, elevated DSBI-RF was found in schizophrenia, bipolar disorder, and autism-spectrum disorders with various anatomic features (cortico-limbic and cortico-cerebellar circuits) [53,54,55].

In a case–control sample of 93 patients with clinically diagnosed major depressive disorder and 93 healthy controls stratified by the presence of complex psychosocial stress (cultural and religious adaptation problems), DSBI-RF showed region-specific patterns that discriminated diagnostic status from psychosocial stress. RF in the amygdala and hippocampus was elevated in participants experiencing psychosocial stress, whereas elevated cortical RF tracked depression diagnosis rather than psychosocial stress [56,57]. DSBI-RF is relevant when only anxiety is present without depression. In a 12-week longitudinal trial of 50 patients with generalized anxiety disorder, algorithm-based modular psychotherapy resulted in parallel clinical and imaging changes in the amygdala (decreased DSBI-RF during treatment) that emphasize the possible role of DSBI-RF in the monitoring of treatment response [58].

In summary, in mood and anxiety disorders, DBSI-RF has been explored as a putative imaging correlate of symptoms and treatment-related changes in single-center, modest-sized cohorts. These findings require replication in independent cohorts.

### 4.4. Summary of Diagnostic Performance and Predictive Value

Studies to date indicate that DBSI-RF may improve diagnostic accuracy in contexts where neuroinflammation is supposed to be a key factor, from MS and AD to mood and anxiety disorders. It might show sensitivity to subtle inflammatory changes, detecting even mild cellular increases (e.g., during complex psychosocial stress). Moreover, by putatively quantifying inflammation, DBSI-RF can have predictive utility, identifying patients at risk of worse outcomes. However, it is often used in conjunction with other metrics; the multiparametric DBSI approach typically yields the best classification, as each metric targets a different pathological feature.

Across published DBSI studies that probe diagnostic or predictive utility, comprehensive performance reporting is limited. Robust metrics (Area Under the Curve (AUC), sensitivity, and specificity) are available for lesion subtype classification in MS (DBSI deep neural network overall concordance: 93.4%; per-class AUCs: 0.98–0.99) and for predicting persistent “black holes” (AUCs: 0.74–0.80) [12,39]. In pediatric obesity, DBSI-RF showed AUCs of 0.80–0.83 in the striatum; however, sensitivities and specificities were not reported [41]. The remaining studies are associative and do not provide robust diagnostic metrics. Extensive, longitudinal, and externally validated studies are needed before positioning DBSI-RF as a clinically ready diagnostic biomarker.

## 5. Strengths and Limitations of DBSI-RF Measurements

### 5.1. Strengths

A significant strength of DBSI-RF is its correlation with cellular inflammation. Unlike conventional diffusion measures (e.g., mean diffusivity or DTI eigenvalues) that conflate various tissue changes, RF captures an increase in cell content [11]. This has been validated by significant correlations with immunobiological markers of microglia/macrophages in both animal models and human tissue, as reviewed above (Table 1). It might provide a noninvasive proxy for neuroinflammation that can be quantified longitudinally in vivo, which is invaluable for monitoring diseases like MS, AD, TBI, metabolic syndrome, and mental disorders, or tracking treatment response from anti-inflammatory therapies to psychological therapies in mood and anxiety disorders.

Beyond RF, it is notable that the multicomponent model of DBSI is an advantage in complex nervous system diseases. It allows RF (inflammation) to be measured simultaneously with metrics for demyelination (e.g., radial diffusivity), axonal injury (axial diffusivity or fiber fraction), and edema (non-restricted water fraction) [11,19]. This multifaceted view reduces interpretative ambiguity. For instance, in an active MS lesion, DBSI can reveal whether a low fractional anisotropy on DTI is primarily due to demyelination or to inflammatory cell influx and edema by examining radial diffusivity versus RF. Studies have shown that DBSI metrics correspond to pathologies more effectively than DTI metrics do [10].

DBSI-RF seems to be sensitive in detecting subtle inflammation that conventional MRI may miss. For example, in the TBI optic neuropathy model, DBSI detected bilateral optic nerve inflammation even when visual function tests only showed unilateral deficits [20]. In occupational manganese exposure, DBSI indicated multifocal white matter inflammation in workers, although routine MRI might appear normal [36]. Finally, in mental disorders, DBSI-RF is sensitive to complex psychosocial stress and changes in anxiety [56]. This high sensitivity means that DBSI-RF could serve as an early biomarker, enabling intervention before significant tissue damage occurs. It may also help track diffuse neuroinflammatory states in neurodegenerative and psychiatric disorders, which are not visible on standard imaging.

The method has good translation. It can be used in animal studies to quantify neuroinflammation, aiding translational research, and then applied in humans with comparable interpretation [11]. The association with outcomes (e.g., lesion chronicity, disease prognosis/subtype in MS, cognitive impairment, and behavioral-psychological symptoms) suggests that it could be used prognostically [16,18,38,56]. Additionally, the ability to distinguish inflammation could guide treatment decisions. Some authors call DBSI combined with artificial intelligence “diffusion histology imaging”, as it approaches pathological specificity comparable to biopsy in the diagnosis of malignant tumors [59].

### 5.2. Limitations

Although the available data are promising, it is essential to consider that the results indicate initial and preliminary diagnostic performance, single-center diagnostic classification, a moderate-to-strong correlation with histology in limited settings, and an exploratory and hypothesis-generating nature for psychiatric applications. Independent replications are indispensable. The number of published studies is small, and the methods are heterogeneous, which precludes the conduct of a systematic review or meta-analysis. A standardized imaging protocol, such as the one used in the United Kingdom Biobank protocol, may significantly facilitate reproducibility (Table 2) [52].

Currently, there is a lack of etiological specificity. While DBSI-RF indicates increased cellularity, it cannot distinguish the cause or type of cells. Neuroinflammation (microglia, macrophages, and lymphocytes) will elevate DBSI-RF, but so will any high-cellularity process. For example, high-grade brain tumors show an elevated RF corresponding to tumor cell density [60]. Thus, an area of high RF could indicate an abscess, a cellular tumor, or inflammation. Within inflammation, DBSI-RF does not distinguish microglial activation vs. lymphocytic infiltration, nor does it reveal whether the inflammation is acute or chronic. In histological studies, advanced immune markers that distinguish cell types have not been used.

DBSI is more demanding than standard DTI [33]. It often entails multiple b-values and numerous diffusion directions to separate components reliably. This means longer scan times and higher data requirements, which can be challenging in routine clinical practice. Though newer optimized protocols are being explored, the availability of DBSI is currently mainly limited to research settings. The need for robust post-processing and modeling is another barrier. Microstructural models can be under-determined and sensitive to acquisition design, including parameter degeneracy and rotationally invariant mapping [61,62]. Thus, while DBSI-RF is powerful, its use as a routine clinical biomarker will depend on simplifying acquisition or incorporating it into MRI vendor software.

DBSI attempts to resolve partial volume confounds, yet the spatial resolution of diffusion MRI is limited. In small structures or cortical gray matter, partial volume effects with cerebrospinal fluid or mixed tissue may affect accuracy. Moreover, if inflammatory cells are present in thin perivascular cuffs or meninges, the voxel-averaged effect may be diluted. The method assumes no water exchange between compartments during diffusion time, and significant exchange could violate model assumptions [10].

The main advantage of DTI is its ability to calculate tensor values that approximate microstructural changes in white matter tracts, which are often affected in neurodegenerative diseases, and can differentiate sophisticated cognitive changes (e.g., prosodic and phonetic aspects of speech) [63]. In contrast, DBSI-RF is an isotropic scalar and, by itself, has no direction information, so one cannot do tensor eigen-decomposition, ellipsoid glyphs, or tractography from RF the way one can do from a DTI tensor. It is possible to render RF as a 3D volume/overlay (e.g., thresholded “inflammation” maps), but RF does not support fiber-orientation–based 3D modeling.

In cases of extremely dense cellular packing, the diffusion signal from restricted water might approach a maximum fraction, potentially limiting the dynamic range. Conversely, in mild inflammation, DBSI-RF changes might be within the range of noise. For instance, in the autopsy MS study, lesions with slight inflammation did not show a significant RF elevation [11]. Additionally, edema often accompanies inflammation. If not adequately accounted for, edema (increasing the “free” water fraction) could mask the RF signal. Overall, DBSI appears to interrogate water diffusion in cellular structures through restricted isotropic diffusion (the cellular infiltrative component) and an additional non-restricted water fraction (edema). However, it is unclear whether this model can also assess sub-diffusion regimes (hindered compartments), as seen in other non-Gaussian models [64].

While many studies support DBSI-RF as a putative inflammation marker, further studies are warranted. Sample sizes in human studies have been modest, and more validation is needed across diverse populations and scanner platforms. Histopathological comparison data in humans are limited, based on case reports or autopsies [11]. Standardizing the threshold that defines “restricted” is also questionable. Some studies used 0.3 μm^2^/ms for in vivo brain tissue, while others used up to 0.6 μm^2^/ms in ex vivo or rodent tissue [11,27,30]. Slight differences in threshold or fitting approach could affect the absolute values across studies. Thus, before DBSI-RF can be adopted widely, consensus on protocols and reference values is needed. Ongoing research, including machine-learning “diffusion histology” approaches, should address these issues by calibrating against histology and optimizing algorithms. These studies and subsequent systematic reviews and meta-analyses should take into account standards for the quality assessments of diagnostic accuracy studies [65].

## 6. Conclusions

DBSI-RF is a promising putative MRI biomarker for neuroinflammation, supported by a convergence of animal and human evidence. It quantitatively reflects the cellular component of pathology, bridging neuroimaging with histopathology. Studies in MS and animal models have established a foundational proof-of-concept: DBSI-RF increases with inflammatory cell influx and correlates with the presence of microglia and macrophages on histology. This has been extended to other contexts like TBI, toxic exposures, and even metabolic disturbances (obesity), neurodegenerative conditions, and mental disorders. The diagnostic accuracy gains may highlight its potential clinical utility, while the ability to predict outcomes could inform prognosis and treatment planning.

Nevertheless, caution is warranted. DBSI-RF is not a standalone “inflammation test” but rather one piece of a multiparametric puzzle. It excels at indicating that cellular proliferation is present, but not why. Thus, it should complement, not replace, other clinical information. Technical challenges in implementation and the need for further validation mean that DBSI-RF remains at the cutting edge of neuroimaging research rather than a routine clinical method. Upcoming studies and technological advancements will determine whether this biomarker can be streamlined for broader use. If so, DBSI-RF holds the promise of more precise diagnosis and monitoring of neuroinflammatory processes across a spectrum of neurological and psychiatric disorders.

## Figures and Tables

**Table 2:** Recommended imaging parameters from the United Kingdom Biobank protocol.

3T MRI scanner MPRAGE (magnetization-prepared rapid acquisition gradient echo) 3D sagittal acquisition FOV (square field of view) = 5256 mm Voxel: 1 × 1 × 1 mm^3^ TI (inversion time) = 5900 ms TE (echo time, shortest) = 3.16 flip angle: 9 degrees no fat suppression full k space acquisition time: 6 min and 50 s acceleration factor: 2 multi-shell approach b1 = 1000 s/mm^2^ b2 = 2000 s/mm^2^ 2 × 2 × 2 mm^3^ 50 diffusion encoding directions for each shell corrections for head motion, outlier slices, and gradient distortion

## Abbreviations

The following abbreviations are used in this manuscript:

Aβ	Amyloid-beta
AD	Alzheimer’s disease
AUC	Area Under the Curve
DAPI	4′,6-diamidino-2-phenylindole
DBSI-RF	Diffusion basis spectrum imaging–derived restricted fraction
DTI	Diffusion tensor imaging
FWI	Free-water imaging
MRI	Magnetic resonance imaging
MS	Multiple sclerosis
NODDI	Neurite orientation dispersion and density
PET	Positron emission tomography
TBI	Traumatic brain injury
RF	Restricted fraction
SANDI	Soma and neurite density image
SANRA	Scale for the Assessment of Narrative Review Articles
